# Bis(4-amino­pyridinium) tetra­chlorido­cobaltate(II)

**DOI:** 10.1107/S1600536809013270

**Published:** 2009-04-18

**Authors:** Samuel Robinson Jebas, A. Sinthiya, B. Ravindran Durai Nayagam, Dieter Schollmeyer, S. Alfred Cecil Raj

**Affiliations:** a Department of Physics, Karunya University, Karunya Nagar, Coimbatore 641 114, India; b Department of Electronics, St. Josephs College, Tiruchirappalli 620 002, India; c Department of Chemistry, Popes College, Sawyerpuram 628 251, Tamilnadu, India; d Institut für Organische Chemie, Universität Mainz, Duesbergweg 10-14, 55099 Mainz, Germany; e Department of Physics, St. Josephs College, Tiruchirappalli 620 002, India

## Abstract

In the title compound, (C_5_H_7_N_2_)_2_[CoCl_4_], the cobalt(II) ion is coordinated by four chloride ions in a slightly distorted tetra­hedral geometry. The crystal packing is stabilized by inter­molecular N—H⋯Cl hydrogen bonding, forming a three-dimensional network. The crystal was a non-merohedral twin emulating tetra­gonal symmetry, but being in fact ortho­rhom­bic.

## Related literature

For the biological activity of 4-amino­pyridine, see: Judge & Bever (2006 ▶); Schwid *et al.* (1997 ▶); Strupp *et al.* (2004 ▶). For related structures, see: Anderson *et al.* (2005 ▶); Chao & Schempp (1977 ▶); Jebas *et al.* (2006 ▶); Zhang *et al.* (2005 ▶). For bond-length data, see: Anderson *et al.* (2005 ▶).
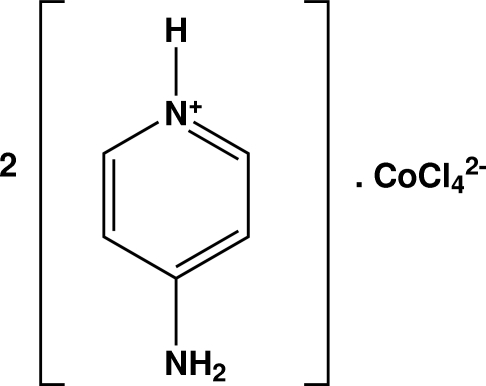


## Experimental

### 

#### Crystal data

(C_5_H_7_N_2_)_2_[CoCl_4_]
*M* *_r_* = 390.98
Orthorhombic, 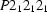

*a* = 15.0051 (12) Å
*b* = 14.9751 (12) Å
*c* = 7.1723 (6) Å
*V* = 1611.6 (2) Å^3^
*Z* = 4
Mo *K*α radiation
μ = 1.72 mm^−1^
*T* = 173 K
0.25 × 0.22 × 0.17 mm

#### Data collection

Bruker APEXII SMART CCD diffractometer
Absorption correction: multi-scan (*SADABS*; Bruker, 2008 ▶) *T*_min_ = 0.650, *T*_max_ = 0.746
45299 measured reflections
3884 independent reflections
3802 reflections with *I* > 2σ(*I*)
*R*_int_ = 0.035

#### Refinement

*R*[*F* ^2^ > 2σ(*F* ^2^)] = 0.021
*wR*(*F* ^2^) = 0.052
*S* = 1.02
3884 reflections
173 parameters
H-atom parameters constrained
Δρ_max_ = 0.25 e Å^−3^
Δρ_min_ = −0.13 e Å^−3^
Absolute structure: Flack (1983 ▶), 1654 Friedel pairs
Flack parameter: −0.12 (2)

### 

Data collection: *APEX2* (Bruker, 2008 ▶); cell refinement: *SAINT* (Bruker, 2008 ▶); data reduction: *SAINT*; program(s) used to solve structure: *SHELXS97* (Sheldrick, 2008 ▶); program(s) used to refine structure: *SHELXL97* (Sheldrick, 2008 ▶); molecular graphics: *SHELXTL* (Sheldrick, 2008 ▶); software used to prepare material for publication: *SHELXTL* and *PLATON* (Spek, 2009 ▶).

## Supplementary Material

Crystal structure: contains datablocks global, I. DOI: 10.1107/S1600536809013270/bt2925sup1.cif

Structure factors: contains datablocks I. DOI: 10.1107/S1600536809013270/bt2925Isup2.hkl

Additional supplementary materials:  crystallographic information; 3D view; checkCIF report

## Figures and Tables

**Table 1:** Hydrogen-bond geometry (Å, °)

*D*—H⋯*A*	*D*—H	H⋯*A*	*D*⋯*A*	*D*—H⋯*A*
N4—H4⋯Cl2^i^	0.88	2.94	3.563 (3)	130
N4—H4⋯Cl3^i^	0.88	2.67	3.335 (3)	134
N7—H7*A*⋯Cl2^ii^	0.84	2.50	3.338 (2)	175
N7—H7*B*⋯Cl1	0.90	2.53	3.387 (2)	158
N11—H11⋯Cl1^iii^	0.87	2.52	3.272 (3)	144
N14—H14*B*⋯Cl2^iv^	0.84	2.64	3.394 (3)	149
N14—H14*A*⋯Cl4	0.90	2.42	3.303 (2)	169

Symmetry codes: (i) 
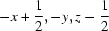
; (ii) 
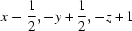
; (iii) 
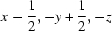
; (iv) 
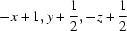
.

**Fig. 1.**
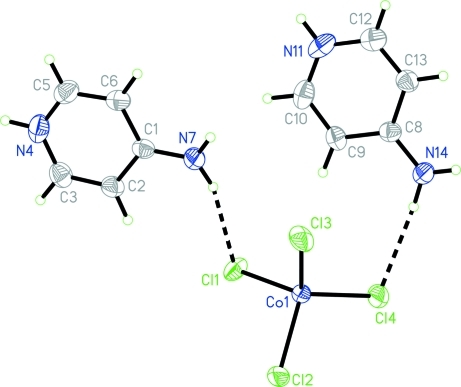
The molecular structure of the title compound, showing 50% probability displacement ellipsoids and the atom numbering scheme. Intramolecular hydrogen bondings are shown as dashed lines.

**Fig. 2.**
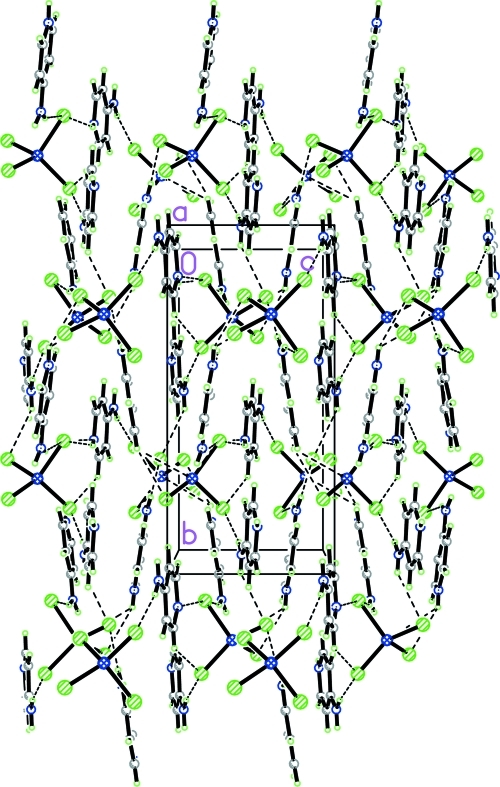
The crystal packing of the title compound, viewed down the a axis, showing the three dimensional network.

## supplementary crystallographic information

### Comment

4-aminopyridine (Fampridine) is used clinically in Lambert-Eaton myasthenic syndrome and multiple sclerosis because by blocking potassium channels it prolongs action potentials thereby increasing transmitter release at the neuromuscular junction (Judge & Bever, 2006; Schwid *et al.*, 1997; Strupp *et al.*, 2004). The structure of 4-aminopyridine has been reported (Chao & Schempp, 1977). Redetermination of the structure of 4-aminopyridine has been reported (Anderson *et al.*, 2005). As a part of our investigation of the reactions of the 4-aminopyridine with metals, we report here the crystal structure of the title compound (I).

The asymmetric unit of (I), consists of two molecules of 4-aminopyridinium cation and a [CoCl_4_]^2-^ anion. The bond lengths and bond angles of the 4-aminopyridinium are comparable with the values reported earlier for the 4-aminopyridine in the its uncomplexed form (Anderson *et al.*, 2005; Chao & Schempp, 1977). Protonation of the atoms N4 and N11 of the 4-aminopyridine leads to the widening of C3–N4–C5 and C10–N11–C12 angles in the pyridine ring to 121.3 (7)° and 120.4 (7)°, compared to 115.25 (13)° in 4-aminopyridine (Anderson *et al.*, 2005). The 4-aminopyridine ring is essentially planar with the maximum deviation from planarity of 0.014 (3) Å for the atoms C8 and N11 respectively.

The anion exhibits distorted tetraedral geometry, with the Co^II^ ion is surrounded by four Cl atoms, with Cl—Co—Cl angles ranging from 106.19 (11)–115.63 (8) Å. The mean Co—Cl bond length, 2.2707 (2) Å, is close to those observed in similar complex (Jebas *et al.*, 2006; Zhang *et al.*, 2005).

The crystal packing (Fig. 2) is consolidated by intermolecular N—H···Cl hydrogen bonding to form a three dimensional network.

### Experimental

4-aminopyridine (0.094 g, 1 mmol) and and CoCl_2_ (0.169 g, 1 mmol) in ethanol (10 ml each) and the solution was stirred well for 3 h. Blue crystals of (I) were obtained by slow evaporation of the solution over a period of one month.

### Refinement

The crystals of (I) crystallized with nearly tetragonal  lattice parameters. It was was not possible to solve and refine the structure in any tetragonal space group but it could be easily solved and refined in orthorhombic space group P2_1_2_1_2_1_. PLATON and the intensity statistic indicate   twinning. Applying the twin instruction TWIN 0 1 0 1 0 0 0 0 -1 with a BASF of 0.340 (1) the R1 value drops to 0.021 (0.095 without TWIN instruction). The nonstandard setting for the orthorhombic cell was kept to simplify the twin matrix.

All the hydrogen atoms were fixed on the calculated positions and allowed to ride on their parent atoms with the C—H = 0.95 Å (aromatic); N—H = 0.84–0.89 Å with *U*_iso_(C) in the range of 1.2U_eq_(C)-1.5U_eq_(N).

### Crystal data

**Table:**

(C_5_H_7_N_2_)_2_[CoCl_4_]	*F*(000) = 788
*M**_r_* = 390.98	*D*_x_ = 1.611 Mg m^−^^3^
Orthorhombic, *P*2_1_2_1_2_1_	Mo *K*α radiation, λ = 0.71073 Å
Hall symbol: P 2ac 2ab	Cell parameters from 9192 reflections
*a* = 15.0051 (12) Å	θ = 2.7–27.8°
*b* = 14.9751 (12) Å	µ = 1.72 mm^−^^1^
*c* = 7.1723 (6) Å	*T* = 173 K
*V* = 1611.6 (2) Å^3^	Block, blue
*Z* = 4	0.25 × 0.22 × 0.17 mm

### Data collection

**Table:**

Bruker APEXII SMART CCD diffractometer	3884 independent reflections
Radiation source: sealed Tube	3802 reflections with *I* > 2σ(*I*)
graphite	*R*_int_ = 0.035
CCD scan	θ_max_ = 28.0°, θ_min_ = 1.4°
Absorption correction: multi-scan (*SADABS*; Bruker, 2008)	*h* = −19→19
*T*_min_ = 0.650, *T*_max_ = 0.746	*k* = −19→19
45299 measured reflections	*l* = −9→9

### Refinement

**Table:**

Refinement on *F*^2^	Secondary atom site location: difference Fourier map
Least-squares matrix: full	Hydrogen site location: inferred from neighbouring sites
*R*[*F*^2^ > 2σ(*F*^2^)] = 0.021	H-atom parameters constrained
*wR*(*F*^2^) = 0.052	*w* = 1/[σ^2^(*F*_o_^2^) + (0.0281*P*)^2^ + 0.361*P*] where *P* = (*F*_o_^2^ + 2*F*_c_^2^)/3
*S* = 1.02	(Δ/σ)_max_ < 0.001
3884 reflections	Δρ_max_ = 0.25 e Å^−^^3^
173 parameters	Δρ_min_ = −0.12 e Å^−^^3^
0 restraints	Absolute structure: Flack (1983), 1654 Friedel pairs
Primary atom site location: structure-invariant direct methods	Flack parameter: −0.12 (2)

### Special details

**Table:**

Geometry. All e.s.d.'s (except the e.s.d. in the dihedral angle between two l.s. planes) are estimated using the full covariance matrix. The cell e.s.d.'s are taken into account individually in the estimation of e.s.d.'s in distances, angles and torsion angles; correlations between e.s.d.'s in cell parameters are only used when they are defined by crystal symmetry. An approximate (isotropic) treatment of cell e.s.d.'s is used for estimating e.s.d.'s involving l.s. planes.
Refinement. Refinement of *F*^2^ against ALL reflections. The weighted *R*-factor *wR* and goodness of fit *S* are based on *F*^2^, conventional *R*-factors *R* are based on *F*, with *F* set to zero for negative *F*^2^. The threshold expression of *F*^2^ > σ(*F*^2^) is used only for calculating *R*-factors(gt) *etc*. and is not relevant to the choice of reflections for refinement. *R*-factors based on *F*^2^ are statistically about twice as large as those based on *F*, and *R*- factors based on ALL data will be even larger. Structure was refined as tetragonal twin with basf=0.34063

### Fractional atomic coordinates and isotropic or equivalent isotropic displacement parameters (Å^2^)

**Table:**

	*x*	*y*	*z*	*U*_iso_*/*U*_eq_	
Co1	0.49922 (2)	0.24481 (2)	0.37955 (6)	0.02580 (7)	
Cl1	0.45208 (4)	0.12712 (5)	0.20160 (11)	0.03485 (16)	
Cl2	0.59477 (5)	0.18722 (5)	0.59594 (11)	0.03872 (17)	
Cl3	0.38406 (6)	0.30281 (5)	0.54058 (11)	0.04194 (19)	
Cl4	0.56931 (5)	0.33990 (5)	0.18373 (14)	0.04232 (17)	
C1	0.19394 (17)	0.06484 (18)	0.2826 (4)	0.0289 (5)	
C2	0.2459 (2)	−0.0132 (2)	0.2552 (4)	0.0337 (6)	
H2	0.3091	−0.0095	0.2540	0.040*	
C3	0.2050 (2)	−0.0935 (2)	0.2307 (4)	0.0379 (7)	
H3	0.2400	−0.1455	0.2116	0.045*	
N4	0.11624 (18)	−0.10010 (17)	0.2331 (3)	0.0413 (6)	
H4	0.0873	−0.1508	0.2248	0.050*	
C5	0.0644 (2)	−0.0280 (2)	0.2604 (4)	0.0403 (7)	
H5	0.0015	−0.0348	0.2631	0.048*	
C6	0.10048 (18)	0.0545 (2)	0.2843 (4)	0.0342 (6)	
H6	0.0630	0.1049	0.3021	0.041*	
N7	0.23233 (15)	0.14446 (15)	0.3030 (4)	0.0382 (5)	
H7A	0.2002	0.1883	0.3330	0.057*	
H7B	0.2909	0.1553	0.2895	0.057*	
C8	0.32310 (18)	0.44022 (18)	0.0283 (4)	0.0289 (5)	
C9	0.3084 (2)	0.34727 (18)	0.0205 (4)	0.0326 (6)	
H9	0.3562	0.3066	0.0389	0.039*	
C10	0.2245 (2)	0.3171 (2)	−0.0139 (4)	0.0398 (7)	
H10	0.2142	0.2546	−0.0204	0.048*	
N11	0.15584 (17)	0.37337 (19)	−0.0389 (3)	0.0418 (6)	
H11	0.1000	0.3568	−0.0377	0.050*	
C12	0.1679 (2)	0.4632 (2)	−0.0254 (4)	0.0382 (7)	
H12	0.1183	0.5021	−0.0385	0.046*	
C13	0.24960 (17)	0.4974 (3)	0.0064 (4)	0.0331 (6)	
H13	0.2575	0.5602	0.0140	0.040*	
N14	0.40431 (15)	0.47315 (16)	0.0593 (3)	0.0375 (5)	
H14A	0.4522	0.4383	0.0767	0.056*	
H14B	0.4130	0.5288	0.0656	0.056*	

### Atomic displacement parameters (Å^2^)

**Table:**

	*U*^11^	*U*^22^	*U*^33^	*U*^12^	*U*^13^	*U*^23^
Co1	0.02267 (19)	0.0223 (2)	0.03247 (13)	0.00081 (16)	−0.00149 (13)	−0.00034 (12)
Cl1	0.0261 (3)	0.0370 (4)	0.0415 (4)	−0.0058 (2)	0.0005 (3)	−0.0129 (3)
Cl2	0.0389 (4)	0.0279 (3)	0.0493 (4)	0.0002 (2)	−0.0190 (3)	0.0004 (3)
Cl3	0.0407 (4)	0.0315 (4)	0.0536 (4)	0.0119 (3)	0.0134 (3)	0.0012 (3)
Cl4	0.0296 (3)	0.0346 (4)	0.0628 (4)	−0.0003 (3)	0.0073 (3)	0.0151 (3)
C1	0.0278 (13)	0.0327 (14)	0.0263 (13)	0.0030 (10)	−0.0015 (11)	0.0023 (11)
C2	0.0296 (15)	0.0333 (15)	0.0383 (15)	0.0032 (11)	0.0010 (11)	0.0018 (13)
C3	0.0486 (17)	0.0302 (14)	0.0349 (15)	0.0031 (12)	−0.0027 (13)	−0.0019 (11)
N4	0.0536 (15)	0.0364 (13)	0.0338 (12)	−0.0155 (11)	−0.0055 (11)	0.0029 (10)
C5	0.0334 (16)	0.0515 (17)	0.0359 (15)	−0.0080 (13)	−0.0017 (12)	0.0069 (12)
C6	0.0289 (14)	0.0408 (16)	0.0329 (14)	0.0033 (11)	−0.0021 (12)	0.0026 (12)
N7	0.0303 (11)	0.0273 (12)	0.0570 (15)	0.0018 (8)	0.0002 (11)	0.0017 (11)
C8	0.0279 (13)	0.0328 (13)	0.0261 (12)	0.0014 (10)	0.0004 (11)	0.0015 (11)
C9	0.0403 (15)	0.0260 (13)	0.0317 (13)	0.0018 (11)	0.0001 (12)	0.0001 (11)
C10	0.0500 (17)	0.0372 (17)	0.0321 (14)	−0.0146 (14)	0.0024 (13)	−0.0031 (12)
N11	0.0305 (12)	0.0627 (17)	0.0322 (11)	−0.0136 (12)	−0.0010 (10)	−0.0001 (12)
C12	0.0342 (15)	0.0499 (18)	0.0304 (14)	0.0011 (12)	0.0018 (12)	0.0038 (13)
C13	0.0307 (14)	0.0327 (16)	0.0357 (14)	0.0041 (10)	0.0040 (13)	0.0027 (11)
N14	0.0279 (11)	0.0295 (11)	0.0550 (15)	−0.0007 (8)	−0.0018 (11)	−0.0008 (10)

### Geometric parameters (Å, °)

**Table:**

Co1—Cl3	2.2527 (8)	N7—H7A	0.8422
Co1—Cl4	2.2597 (8)	N7—H7B	0.8984
Co1—Cl2	2.2822 (8)	C8—N14	1.333 (3)
Co1—Cl1	2.2880 (8)	C8—C13	1.405 (4)
C1—N7	1.332 (3)	C8—C9	1.410 (4)
C1—C6	1.411 (4)	C9—C10	1.360 (4)
C1—C2	1.419 (4)	C9—H9	0.9500
C2—C3	1.361 (4)	C10—N11	1.344 (4)
C2—H2	0.9500	C10—H10	0.9500
C3—N4	1.335 (4)	N11—C12	1.360 (4)
C3—H3	0.9500	N11—H11	0.8737
N4—C5	1.345 (4)	C12—C13	1.348 (4)
N4—H4	0.8772	C12—H12	0.9500
C5—C6	1.359 (4)	C13—H13	0.9500
C5—H5	0.9500	N14—H14A	0.8967
C6—H6	0.9500	N14—H14B	0.8440
			
Cl3—Co1—Cl4	115.64 (3)	C1—N7—H7A	118.5
Cl3—Co1—Cl2	106.20 (4)	C1—N7—H7B	124.9
Cl4—Co1—Cl2	111.62 (3)	H7A—N7—H7B	116.6
Cl3—Co1—Cl1	110.24 (3)	N14—C8—C13	120.7 (3)
Cl4—Co1—Cl1	106.41 (4)	N14—C8—C9	121.0 (2)
Cl2—Co1—Cl1	106.41 (3)	C13—C8—C9	118.3 (3)
N7—C1—C6	121.8 (2)	C10—C9—C8	118.7 (3)
N7—C1—C2	121.0 (3)	C10—C9—H9	120.7
C6—C1—C2	117.2 (3)	C8—C9—H9	120.7
C3—C2—C1	119.8 (3)	N11—C10—C9	121.7 (3)
C3—C2—H2	120.1	N11—C10—H10	119.1
C1—C2—H2	120.1	C9—C10—H10	119.1
N4—C3—C2	121.0 (3)	C10—N11—C12	120.6 (3)
N4—C3—H3	119.5	C10—N11—H11	123.8
C2—C3—H3	119.5	C12—N11—H11	114.0
C3—N4—C5	121.2 (3)	C13—C12—N11	120.6 (3)
C3—N4—H4	123.8	C13—C12—H12	119.7
C5—N4—H4	114.8	N11—C12—H12	119.7
N4—C5—C6	121.2 (3)	C12—C13—C8	120.1 (3)
N4—C5—H5	119.4	C12—C13—H13	120.0
C6—C5—H5	119.4	C8—C13—H13	120.0
C5—C6—C1	119.6 (3)	C8—N14—H14A	122.7
C5—C6—H6	120.2	C8—N14—H14B	121.0
C1—C6—H6	120.2	H14A—N14—H14B	116.3
			
N7—C1—C2—C3	−178.6 (3)	N14—C8—C9—C10	−179.2 (3)
C6—C1—C2—C3	0.5 (4)	C13—C8—C9—C10	2.2 (4)
C1—C2—C3—N4	−0.4 (4)	C8—C9—C10—N11	−0.5 (4)
C2—C3—N4—C5	−0.2 (5)	C9—C10—N11—C12	−1.8 (4)
C3—N4—C5—C6	0.8 (4)	C10—N11—C12—C13	2.5 (5)
N4—C5—C6—C1	−0.7 (4)	N11—C12—C13—C8	−0.8 (4)
N7—C1—C6—C5	179.1 (3)	N14—C8—C13—C12	179.8 (3)
C2—C1—C6—C5	0.0 (4)	C9—C8—C13—C12	−1.5 (4)

### Hydrogen-bond geometry (Å, °)

**Table:**

*D*—H···*A*	*D*—H	H···*A*	*D*···*A*	*D*—H···*A*
N4—H4···Cl2^i^	0.88	2.94	3.563 (3)	130
N4—H4···Cl3^i^	0.88	2.67	3.335 (3)	134
N7—H7A···Cl2^ii^	0.84	2.50	3.338 (2)	175
N7—H7B···Cl1	0.90	2.53	3.387 (2)	158
N11—H11···Cl1^iii^	0.87	2.52	3.272 (3)	144
N14—H14B···Cl2^iv^	0.84	2.64	3.394 (3)	149
N14—H14A···Cl4	0.90	2.42	3.303 (2)	169

Symmetry codes: (i) −*x*+1/2, −*y*, *z*−1/2; (ii) *x*−1/2, −*y*+1/2, −*z*+1; (iii) *x*−1/2, −*y*+1/2, −*z*; (iv) −*x*+1, *y*+1/2, −*z*+1/2.
